# Can human papillomavirus vaccination during pregnancy result in miscarriage and stillbirth? A meta-analysis and systematic review

**DOI:** 10.18332/ejm/161793

**Published:** 2023-04-29

**Authors:** Rana Dousti, Leila Allahqoli, Asiye Ayar Kocaturk, Sevil Hakimi

**Affiliations:** 1 Faculty of Nursing and Midwifery, Tabriz University of Medical Science, Tabriz, Iran; 2 Midwifery Department, Ministry of Health and Medical Education, Tehran, Iran; 3 School of Health Sciences, Istanbul Medipol University, Istanbul, Turkey; 4 Faculty of Nursing and Midwifery, Research Center of Psychiatry and Behavioral Sciences, Tabriz University of Medical Sciences, Tabriz, Iran

**Keywords:** vaccination, HPV, pregnancy, miscarriage, stillbirth

## Abstract

**INTRODUCTION:**

Contradictory results regarding the safety of human papillomavirus (HPV) vaccination during pregnancy have been obtained, which has cast doubt on the use of this method. This review and meta-analysis were conducted to evaluate the safety of HPV vaccination during pregnancy.

**METHODS:**

Complying with the inclusion and exclusion criteria, we searched Web of Science, Scopus, Medline, EMBASE, PubMed and Google Scholar databases for articles published in the past decade using the following keywords: ‘papilloma human virus’, ‘HPV vaccine’, ‘pregnancy’ and ‘safety and prevention’. The minimum report quality of the articles was 16 based on the STROBE checklist.

**RESULTS:**

Seven articles were included in the study, three of which were included in the meta-analysis, and the rest were reviewed systematically. The results of the meta-analysis showed that vaccination against HPV during pregnancy or around this period does not increase the risk of miscarriage (RR=2.01; 95% CI: 0.66–6.13) and stillbirth (RR=2.02: 95% CI: 0.65–6.27). No significant difference between miscarriage and stillbirth was observed in women vaccinated against HPV versus those not vaccinated.

**CONCLUSIONS:**

The study of 1380424 individuals showed that HPV vaccination during pregnancy is better postponed until after this period. However, no significant evidence was found to indicate that vaccination was dangerous and unsafe during pregnancy. Further studies are needed to draw a more definitive conclusion.

## INTRODUCTION

HPV is one of the largest families of infectious viruses and the most common type of sexually transmitted infection^1^. HPV is the most prevalent sexually transmitted infection globally^2^, it is often asymptomatic and is self-limiting. The virus has double-stranded DNA that infects the skin and mucous membranes and can cause extensive symptoms^3^. The majority of HPV infected cases cure spontaneously. Evidence indicates that virus is not detectable within two years in more than 90% of cervical HPV infection cases. However, infection with one of thirteen types of oncogene HPV types can be carcinogenous^4,5^. HPV has a strong association and is the main cause of cervical cancer, a cancer with a high fatality rate among women^6^. Other than cervical cancer, it is known as a cause of vaginal, vulvar, penile, rectal, as well as oropharyngeal cancers^4^.

HPV primarily affects youth aged <25 years. The prevalence of HPV infection is estimated to be nearly 12% among young adult women with normal cervical cytology^7^. Mesher et al.^8^ in their study in England, showed that among positive swab samples, 19% were positive for HPV type 16 and 6.5% for HPV type 18. Similarly, So et al.^9^ in South Korea, examined 986 healthy women and identified that the prevalence of HPV was 33.7%9. Results of the study by Mbulawa et al.^10^, revealed that HPV prevalence among healthy South African women was approximately 37%. This prevalence was higher among young (18–25 years) women.

Because of the significant side effects that the virus may have on women and even on men, it is recommended that women aged 9–26 years be vaccinated against the virus^1^. According to the World Health Organization (WHO), the target group for HPV vaccination is adolescent girls (aged 9–14 years). Vaccination has been recommended for girls aged <15 years as two doses (0 and 6 months). For girls aged ≥15 years, three doses (0, 2 and 6 months) have been recommended. The upper age limit for HPV vaccination is 26 years^11,12^.

HPV vaccination is not recommended during pregnancy^13^, but since pregnancy tests are not routinely performed before vaccination, some pregnant women may inadvertently be vaccinated against HPV^14^. A 2009 community-based clinical trial showed a link between spontaneous miscarriage in women who had been vaccinated against HPV^15^. Due to the adverse effects of HPV vaccination, screening programs on individuals receiving the vaccination are being conducted in some countries^14^. On the other hand, in some other studies, no adverse effects of HPV vaccination have been observed, and it is only recommended that this vaccination be postponed until after pregnancy^16^. To date, there is inconclusive evidence of the impact of HPV vaccination during pregnancy^17^; therefore, some researchers vaccinate patients during pregnancy^18^. Thus, this systematic review and meta-analysis were conducted to review studies on HPV vaccination in pregnant women.

## METHODS

This study was conducted during 2020 in Tabriz University of Medical Sciences, Iran. The review looked at the safety of the HPV vaccine in pregnant women. It was searched in databases including Web of Science, Scopus, Medline, EMBASE, PubMed, and Google Scholar; all articles published during 2009 to 2019 were entered into this review study by observing the inclusion and exclusion criteria. Keywords used to search these databases included ‘human papillomavirus’ or ‘HPV vaccine’ and ‘pregnancy’, ‘immunity’, and ‘prevention’ and ‘abortion’ or ‘miscarriage’ and ‘stillbirth’. The criteria for entering the study included the keywords mentioned in the title and the keywords of the article, and the criteria for excluding the study comprised studies without full text, abstracts presented at conferences (without full text), and articles not fully related to the subject. References of the retrieved articles were also reviewed.

Vaccinated women were women who had been injected with at least one dose of one of the two types of HPV vaccine (bivalent or quadrivalent) six weeks before the last menstrual period (LMP) or during pregnancy. In this study, miscarriage and stillbirth were the two studied outcomes. Miscarriage was considered fetal loss up to 23 weeks of gestation, and stillbirth was defined as intrauterine death after 23 weeks of gestation.

The quality of article reporting was assessed based on the STORBE checklist. This checklist has 22 items that consist of six general sections; these six sections include the title and abstract (1 item) Introduction (2 items), Methods (9 items), Results (5 items), Discussion (4 items) and other data includes the source of funding (1 item)^19^. Out of the 22 items on this checklist, 18 items are generally used in all types of studies, and four items are considered professionally depending on the type of study. Accordingly, articles unrelated to the purpose of the study, duplicate articles, and abstracts without sufficient evidence and having a score <16, according to the STROBE criterion, were excluded from the study.

All steps were performed by two researchers (RD and SH) at a time interval of two weeks so that after searching for articles and combining them in one system, RD examined the articles individually and SH after two weeks from the completion of the first researcher’s work began reviewing the articles. If there was a disagreement between the two researchers in entering or leaving out the article or articles, RN assisted them. Risk ratio was calculated for cohort articles that were entered into the meta-analysis. Risk ratio was calculated separately for the outcomes of miscarriage and stillbirth.

Chi-squared and I^2^ statistics were used to calculate heterogeneity. The significance level was considered to be 0.05. Statistical analysis was performed using the Review Manager (Revman) version 5.2. Significant heterogeneity was observed for both miscarriage and stillbirth outcomes.

**Table 1:** The results of the included studies on the impact of HPV vaccination during pregnancy and miscarriage/stillbirth

*Authors Year*	*Study type*	*STROBE score*	*Sample size*	*Vaccination time*	*Results*
Faber et al.^24^ 2019	Cohort	20	522705	Four weeks before LMP and during pregnancy	Vaccination was not associated with an increase in spontaneous miscarriage.
Kharbanda et al.^20^ 2018	Retrospective cohort	20	2800	Six weeks before LMP and during pregnancy	Vaccination does not cause spontaneous miscarriage.
Scheller et al.^25^ 2017	Cohort	20	581550	During pregnancy	Vaccination was not associated with an increase in spontaneous miscarriage.
Panagiotou et al.^23^ 2015	Cohort	20	7466	Four weeks before LMP and during pregnancy	Vaccination was not associated with an increase in spontaneous miscarriage.
Goss et al.^21^ 2015	Cohort	20	1752	Four weeks before LMP and during pregnancy	Vaccination was not associated with an increase in spontaneous miscarriage.
Baril et al.^16^ 2015	Cohort	20	161849	Thirty days before and 90 days after LMP	Vaccination was not associated with an increase in spontaneous miscarriage.
Dana et al.^22^ 2009	Cohort	18	517	Four weeks before LMP and during pregnancy	Vaccination was not associated with an increase in spontaneous miscarriage and stillbirth.

LMP: last menstrual period.

## RESULTS

### Selection of eligible studies and study characteristics

**Figure 1:**
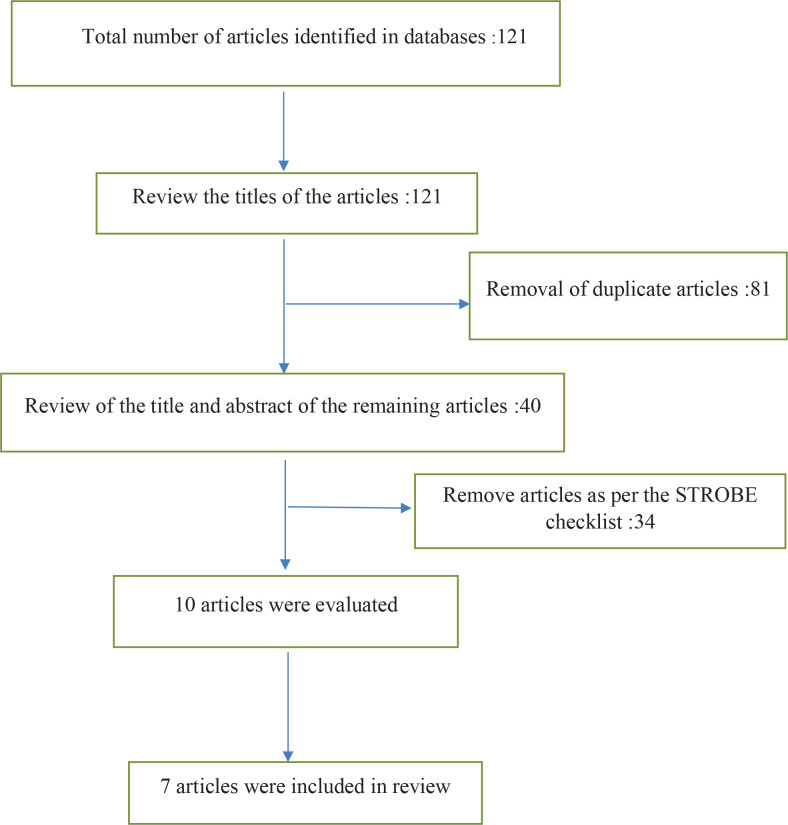
Article selection flowchart

In the initial search, the total number of articles found was 121, of which 81 were duplicate articles, and 40 articles were reviewed based on the STORBE checklist, of which only ten articles remained and were reviewed. Three of those articles were clinical trials and seven articles were descriptive (cohort) studies. Finally, six articles were entered into this systematic review and meta-analysis (Figure 1).

Kharbanda et al.^20^, in their retrospective cohort study of 2800 pregnancies between 2008 and 2014, found that 35% had received at least one dose of the quadrivalent vaccine around pregnancy (6 weeks before LMP), and 32% of them had received it during pregnancy. The risk of miscarriage did not increase in women who had received the vaccine during pregnancy (adjusted hazard ratio, AHR=1.10; 95% CI: 0.81–1.51) and around pregnancy 1.07 (95% CI: 0.81–1.41). In general, the results of this study showed that vaccination during or around pregnancy does not increase the risk of miscarriage.

Goss et al.^21^ conducted a prospective study involving 1752 pregnant women. The results showed that pregnancy outcomes were above 96% without any abnormalities or complications; the overall rate of spontaneous miscarriage was 6.7 per 100 outcomes (95% CI: 5.5–8.2). The mortality rate was 0.8 per 100 outcomes (95% CI: 0.4–1.4). Although the prevalence of spontaneous miscarriage and stillbirth in the population receiving the quadrivalent HPV vaccine during pregnancy did not differ from the general population, researchers do not recommend HPV vaccination during pregnancy.

Dana et al.^22^ in a cohort study examining the results (517 pregnancies) of HPV vaccination in women and infants in the United States, Canada, and France, found that the overall rate of spontaneous miscarriage was 6.9 per 100 outcomes. They concluded that although the incidence of spontaneous miscarriage was not higher in those who were vaccinated during pregnancy than in those who were not vaccinated, it was better to postpone HPV vaccination until after pregnancy.

Panagiotou et al.^23^ in a long-term cohort study, assessed the potential effect of the HPV vaccine on miscarriage in Costa Rica. Of 7466 women, 3727 received three doses of bivalent HPV or hepatitis A vaccines. The results of the study showed no evidence of an increased risk of miscarriage.

**Figure 2:**
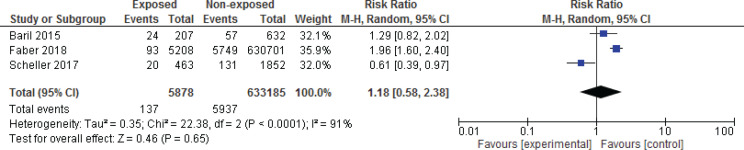
Meta-analysis of the impact of HPV vaccination during pregnancy and spontaneous miscarriage. Random effect analysis was used for the meta-analysis

Among the studies evaluated, three articles had the necessary criteria to enter the meta-analysis^16,24,25^. Figures 1 and 2 show the meta-analysis results for spontaneous miscarriage and stillbirth, respectively. In these three studies, all of which were cohorts, the effect of the vaccine on spontaneous miscarriage and stillbirth was investigated by risk ratio calculation. The results of the meta-analysis showed that HPV vaccination during pregnancy did not increase the risk of miscarriage (RR=2.01; 95% CI: 0.66–6.13) and stillbirth (RR=2.02; 95% CI: 0.65–6.27).

**Figure 3:**
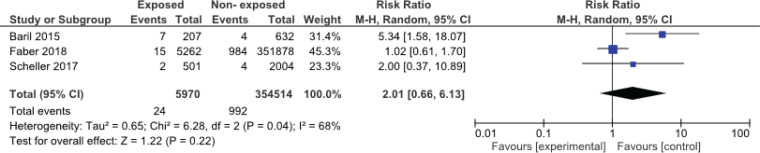
Meta-analysis of the impact of HPV vaccination during pregnancy and stillbirth. Random effect analysis was used for the meta-analysis

## DISCUSSION

This review study aimed to investigate the effects of vaccination against HPV on spontaneous miscarriage and stillbirth. Our review revealed that none of the studies showed an increase in spontaneous miscarriage and stillbirth following vaccination; studies have even shown no statistically significant differences between vaccinated and non-vaccinated groups regarding these two outcomes.

Cervical cancer is the 4th most prevalent cancer among women cancers in the world, with a high fatality rate. Implementation of the HPV vaccine is probably the most effective way to reduce the burden of cervical cancer^26^. HPV vaccination is recommended for all girls/women aged 9–26 years through national immunization programs. Moreover, a ‘catch-up vaccine’ for unvaccinated adults with high-risk behaviors has also been suggested^27^. At present, three types of HPV vaccine are available, the bivalent vaccine is an effective vaccine against HPV types 16 and 18. Quadrivalent vaccine can cover types 6, 11, 16, 18 and the nonavalent vaccine covers even more types of HPV: 6, 11, 16, 18, 31, 33, 45, 52, and 58^28^. The bivalent, quadrivalent and the nonavalent HPV vaccines are recombinant. They contain virus-like particles which promote an immune response more severe than natural infection^29^.

While a considerable proportion of vaccine recipients are women in reproductive age, HPV vaccination may be given during or immediately prior to pregnancy for a variety of reasons. Current guidelines indicate that HPV vaccination during pregnancy, along with its benefits, may be associated with side effects and, therefore, recommend postponing it to the postpartum period^30-32^. WHO and some vaccine manufactures recommend avoiding vaccination during pregnancy^33^.

However, studies examining the results of vaccinations during pregnancy in different countries have not confirmed the reported complications of HPV vaccination, to which our study results are in accordance. Angelo et al.^23^ and Skinner et al.^34^ did not find any relation between HPV vaccine and miscarriage among women who received HPV vaccine between 4 weeks before and 45 days after conception. Moro et al.^35^ carried out a pooled analysis consisting of five randomized trials in which 1796 women were vaccinated with HPV quadrivalent during pregnancy and did not identify differences for miscarriage between vaccinated and unvaccinated women. Moreira et al.^36^, conducted a study to assess the safety of nonavalent HPV vaccine. The study was a phase III clinical trial on men and non-pregnant women in which the frequency of miscarriage in the group of women who received the nonavalent HPV vaccine did not different with women who take quadrivalent one. Miscarriage rate was in normal range within the two groups^36^. Lipkind et al.^37^ in their study showed that administration of quadrivalent HPV vaccine during preconception time or within pregnancy is not related with increased preterm birth and birth defects.

Evidence suggests that the risk of complications in the vaccine recipient group is higher (not significant). By assessment of the results of various studies that included 1380424 pregnant vaccinated women, we found that vaccination during pregnancy is not dangerous. Vaccination during pregnancy has been reported to be without any problems; however, in all these studies, it has been recommended to delay the vaccination to the postpartum period. WHO in its position paper about HPV vaccination, has recommended avoiding it during pregnancy. It also notes that, if a woman becomes pregnant after injection with a HPV vaccine, completion of vaccination should be postponed until after pregnancy. Termination of pregnancy is not necessarily due to HPV vaccination^12^.

### Limitations

Our results are restricted by a number of limitations. The number of studies that could be included in the meta-analysis was small, and the research team was forced to analyze the rest of the studies systematically and not perform subgroup analyses. The results of the meta-analysis showed high heterogeneity of studies, while publication bias was not assessed. Different populations and different definitions from the time of miscarriage and stillbirth are the possible causes of high heterogeneity, especially in results regarding spontaneous miscarriage.

## CONCLUSIONS

The results of the study showed that miscarriage and stillbirth after receiving HPV vaccine during pregnancy were not significantly higher than in comparison populations, however, it is recommended to pause vaccination until the post-pregnancy period, until further research becomes available. Considering the burden of HPV, it is essential to stress the need for vaccination among young adults.

## Data Availability

The data supporting this research are available from the authors on reasonable request.
